# Annotations

**Published:** 1911-05-20

**Authors:** 


### ANNOTATIONS.

### The Medical Defence Union Report.

So numerous and of such variety are the difficulties which may and do confront practitioners, that the annual report of the Defence Union is always full of interesting reading; for it is an epitome, as it were, of all the harassments of private and consulting practice. In former years we have often availed ourselves of the material provided by these reports to point the moral which is by this time becoming fairly well impressed on the profession—namely, that the subscription to a medical defence society is the very best investment that any doctor can possibly make. Notwithstanding the steadily increasing prestige of these societies, and of the Defence Union especially, and notwithstanding the immense number of speculative and swindling actions which are immediately dropped when it is discovered that the Union will accept service on behalf of its members, the past year was notable for the unusual amount of costly litigation undertaken on behalf of members of the Union. It is satisfactory to be able to add that of the 183 cases in which the Union's solicitor was required, in only two was the result unfavourable. As a result of this unusually heavy cause list, the Union was able to add only about £40 to its accumulated funds, a much smaller sum than is commonly the case; but the Society has reached such a state of financial stability that this small profit on the year's working is of little importance compared with the great value to the profession of the protection which has been so frequently extended during 1910 to the members. As usual, the Union was awarded costs against a large number of those who brought actions against its members; and, as usual, not a penny of these costs was recoverable. In three cases alone the Union was thus awarded £800 in costs. In one such case the plaintiff gave evidence of a considerable income, while endeavouring to show that substantial damages should be given him owing to his invalidity; but when it came to paying costs it appeared he had almost nothing. A prosecution for perjury might possibly do something towards stopping this scandalous state of affairs.

### The Royal Academy.

At this year's Royal Academy exhibition, Sir Henry Morris represents the medical profession among the portraits. If the visiting doctor wants any other subject intimately connected with his profession he should go and look at No. 1665 in the Architectural room and see the design for the new examination hall where his successors will be examined. If he were ever " up " for medicine on a hot day in July he will remember the noise of trams and 'buses coming in through the windows and mingling with the râles and rhonchi in his stethoscope, and will envy the future quietude of the examinees in Queen's Square. The water-colour room this year, as usual, contains many delightful small pieces. There is always a holiday feeling in this room, with its little bits of quaint foreign towns, little sketches of country villages, with no problem pictures, no ceremonial portraits, nothing to suggest London but little bits of the river below London Bridge. Of the set portraits of children we may single out Alexandra Franzisha Euphemia Norman-Neruda by Mowat Loudoun (No. 186). Mr. Orchardson's " Problem in White " (No. 310) must touch the heart of anyone who has seen an anxious mother bend over her sick child, while the skilful arrangement of the shades of white—the mother's dress and the hangings of the bed—with the one touch of colour on the sick babe's cheek, must equally appeal to the eye. In his " Golden Dustman " (No. 22) Mr. George E. Butler has given us all imaginable children jumbled together: the nipper from the streets, the babe that cannot walk, rich children and poor children, all just dropping off to sleep. But no one should leave this year's Academy without going into the Lecture room and seeing two small busts of children—" Little Anna " of Frances Burtison (No. 1928), and best of all " The Dawn of Thought " (No. 1923) by S. W. Ward Wallis. There is arising in the Academy, or being infused into it, a school of light and shade that promises well for the future of English painting. Mr. George Clausen has given us another interior of a barn in No. 59, similar in subject to his Diploma work. When we say that the picture made us think of Pieter van der Hooch at the Wallace Collection we can give Mr. Clausen no higher praise. Mr. John Lavery is a welcome addition to the list of Associates, and his picture of Tangier by Night (No. 57) is another representative of his school. Yet other examples are the works of Mr. Arthur Hacker. This artist has passed from the nude to a delineation of street scenes with the happiest result. He gives us three such—his Diploma work " A Wet Night at Piccadilly Circus " (No. 28), " Matinée Afternoon, Piccadilly Circus " (No. 376), and a view of Trafalgar Square called " King Charles' Day " (No. 180). In each the effect of light on the dingy buildings and pavements of London is masterfully depicted.

### Is Insanity Decreasing?

It might at first sight appear satisfactory that the reports of the lunacy officials for the three Kingdoms for 1910 are unanimous in declaring a reduced rate of accumulation of the officially recognised insane. Before expressing satisfaction, however, it is well to decide whether the reduced first admissions are due to cases of mental disorder really being less numerous, or to such cases being treated to a larger extent outside asylums. Statistics are not available to show whether this latter practice is becoming more common, though the English report shows that 9.3 per cent. of first admissions had been previously treated for first attacks outside asylums. It is probable that of recent years private treatment has increased with regard to such cases, while many receive attention first in workhouses both in England and Scotland. Until definite information and reliable statistics are procurable outside asylums, it is impossible to come to any satisfactory conclusion with regard to the actual frequency of the occurrence of insanity.

